# A Robust Step Detection Algorithm and Walking Distance Estimation Based on Daily Wrist Activity Recognition Using a Smart Band

**DOI:** 10.3390/s18072034

**Published:** 2018-06-25

**Authors:** Duong Trong Bui, Nhan Duc Nguyen, Gu-Min Jeong

**Affiliations:** School of Electrical Engineering, Kookmin University, 861-1 Jeongnung-dong, Seongbuk-gu, Seoul 136-702, Korea; buitrongduong@kookmin.ac.kr (D.T.B.); nhannd@kookmin.ac.kr (N.D.N.)

**Keywords:** step detection, walking distance, wrist activity, smart band

## Abstract

Human activity recognition and pedestrian dead reckoning are an interesting field because of their importance utilities in daily life healthcare. Currently, these fields are facing many challenges, one of which is the lack of a robust algorithm with high performance. This paper proposes a new method to implement a robust step detection and adaptive distance estimation algorithm based on the classification of five daily wrist activities during walking at various speeds using a smart band. The key idea is that the non-parametric adaptive distance estimator is performed after two activity classifiers and a robust step detector. In this study, two classifiers perform two phases of recognizing five wrist activities during walking. Then, a robust step detection algorithm, which is integrated with an adaptive threshold, peak and valley correction algorithm, is applied to the classified activities to detect the walking steps. In addition, the misclassification activities are fed back to the previous layer. Finally, three adaptive distance estimators, which are based on a non-parametric model of the average walking speed, calculate the length of each strike. The experimental results show that the average classification accuracy is about 99%, and the accuracy of the step detection is 98.7%. The error of the estimated distance is 2.2–4.2% depending on the type of wrist activities.

## 1. Introduction

In recent years, human activity recognition and pedestrian dead reckoning using inertial sensor-based wearable devices have received much attention of researchers to support human life [1,2,3,4,5,6,7,8,9,10]. Using more wearable devices is synonymous with deploying more sensors. However, this is inconvenient for users if they simultaneously use many devices and perform various daily activities [11]. The main problem is that researchers must find a robust algorithm with high performance using the smallest number of sensors to provide the most convenience for users.

Some studies estimated the walking distance by considering a small number of user’s walking modes and device poses [5,10,12]. Phuc et al. [12] presented the precise stride counting-based method to estimate the walking distance using insole sensors. The insole sensors consisted of a triaxial inertial sensor and eight pressure sensors. The authors estimated the traveling distance based on the number of strides extracted from the phase information. However, they only considered the walking distance estimation of normal walking on flat ground. Lee et al. [5] introduced a robust step detection algorithm for three step modes and seven device poses of the smartphone. The step detection used an adaptive magnitude and temporal thresholds which addressed the transition among step modes or device poses and the time-varying pace of human walking or running problems. The developed method can detect the number of steps for any combination of step mode and device pose. Ho et al. [10] developed a method of walking distance estimation based on an adaptive estimator of the step length and robust step detection. The presented method successfully estimated the traveling distance at three speed levels and four different distances. Furthermore, the step-length estimator, which was an improvement of Weinberg equation [13], used an adaptive *K*-value as a linear regression model.

In common approaches, all processed activities data are directly fed to an adaptive step detector without classifying the performing activities [5,6]. However, it is more effective if the activities are classified because the thresholds of the acceleration values depend on the type of activities. To achieve high accuracy in estimating the traveling distance with various actions, e.g., texting, calling, and swinging, some studies [9,14,15] addressed the problem by implementing classifiers or improving the step detection algorithms before estimating the distance. Susi et al. [9] proposed an adaptive step detection by analyzing the characteristics of the gait cycle, which included the hand motion and carrying-mode difference of a pedestrian using a smartphone. The authors detected the motion modes, e.g., swinging, texting, phoning, bag and irregular motion, before applying the step detection algorithm on the collected inertial signals. Renaudin et al. [14] estimated the step length using a handheld sensor, which was an extended idea from [9]. The presented step detection algorithm used the step frequency, height of the pedestrian, and three variables to estimate the step frequency of non-body fixed sensors. Zhang et al. [15] designed an inertial pedestrian navigation system (IPNS) based on the improvement of the step mode and device pose algorithm using a low cost hand-held device. The step detection algorithm addressed the over-counting and under-counting errors by implementing a support vector machine that was used to recognize step modes and device poses.

The aforementioned studies do not account for the errors caused by the classifiers and step detectors. Therefore, the step detector can make a serious error, where it attempts to detect the steps of a calling or texting activity, which is classified as hand swinging and vice versa. The system can give an exception message when we do not handle these errors. Specifically, the accuracy rates of the walking distance estimator significantly decrease. Thus, in the paper, we propose a new method that uses a smart band to estimate the walking distance based on a robust step detection and an adaptive step length estimation for five daily wrist activities during walking: phone texting, phone calling, hand in pocket, suitcase carrying and hand swinging. The performance of step detection and traveling distance estimation can be improved by applying classifiers, robust step detectors, and the error feedback technique. The activity samples are classified and labeled by support vector machine (SVM) classifiers. A 2-s window of the preprocessed data is used to obtain features that are fed to the classifiers. The step detector used adaptive thresholds each activity. Basically, activity samples can be classified two times before being fed into the step detectors. The movement distances are estimated by summing the length of all walking steps. Furthermore, the step length equation is constructed based on a non-parametric regression of an average magnitude of tri-axial velocities and a set of variables. The contributions of this paper are as follows:

- Developing a hierarchy framework of the walking distance estimation for five daily living activities: phone texting; phone calling; hand in pocket; suitcase carrying; hand swinging.
- Proposing a robust step detection algorithms using an adaptive threshold.
- Improving the step detectors and traveling distance estimators using error feedback.
- Developing the step-length estimation based on non-parametric regression.
- Estimating and comparing the performance of each walking distance estimator with various activities and speed levels.

This paper is organized as follows. In Section 2, we describe the hierarchical framework of the walking distance estimation in details. Section 3 shows the results of our method in three parts: activity classification, step detection, and walking distance estimation. Finally, in Section 4, we conclude the paper and provide directions for future works.

## 2. Walking Distance Estimation Based on Wrist Activity Recognition

### 2.1. Proposed Hierarchical Framework of the Walking Distance Estimation

**Figure 1:**
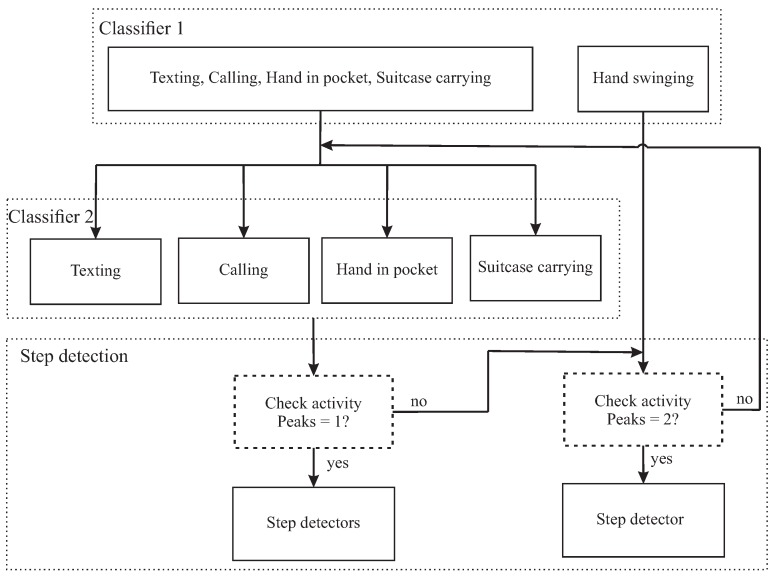
Brief structure of activity classification-based step detection.

**Figure 2:**
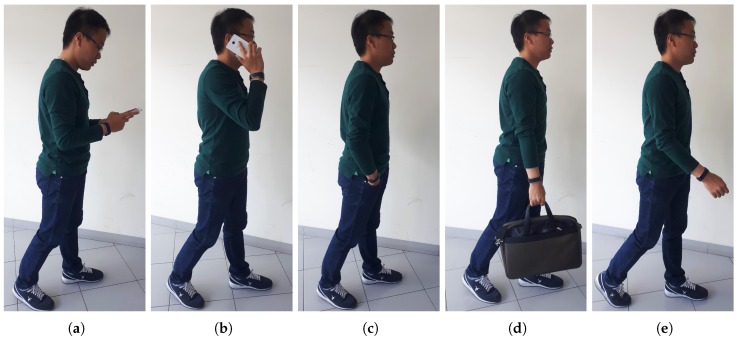
Five daily hand activities during walking: (**a**) texting; (**b**) calling; (**c**) hand in pocket; (**d**) suitcase carrying; (**e**) swinging.

Our objective is to improve the performance of the walking distance estimation using two layers of activity classification (Figure 1). The first layer divides five activities into two groups. Group 1 contains phone calling, texting, suitcase carrying and hand in pocket during walking; group 2 contains hand swinging during walking. The second layer separately classifies the activities in group 1. The five daily wrist activities are described in Figure 2. The characteristic of the generated acceleration signal on the wrist during walking depends on type of user’s wrist activities. Each activity has a different threshold value to detect a peak and a valley of the acceleration signal. Therefore, the step detection algorithm performs more effectively if it knows the type of data that is being processed.

**Figure 3:**
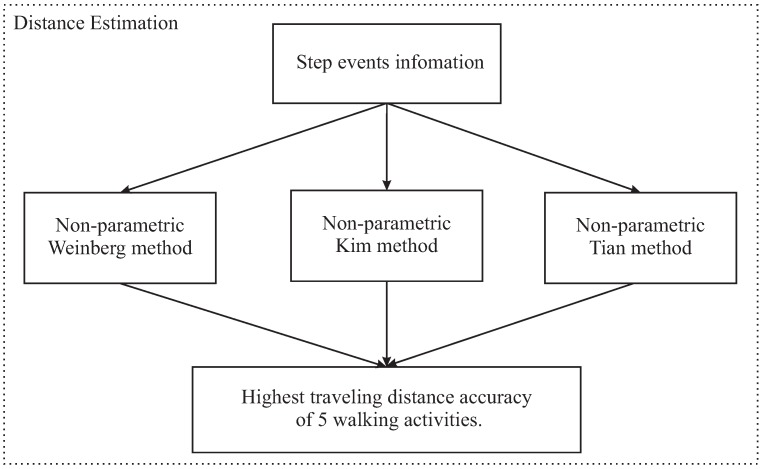
Brief structure of step detection-based distance estimation.

In the step detection phase, the number of peaks between two valleys is checked to make a final decision about the type of activity being processed using the relationship among a step event, a peak and a valley of the filter acceleration signal. Therefore, the step detection phase can detect the misclassification activities and return it to the previous layer. After the activity is determined, an adaptive threshold algorithm is applied to detect the step events. Once the step events are identified, three non-parametric step length models are applied (Figure 3). For each class of activities, only the highest distance accuracy of those three models is considered.

**Figure 4:**
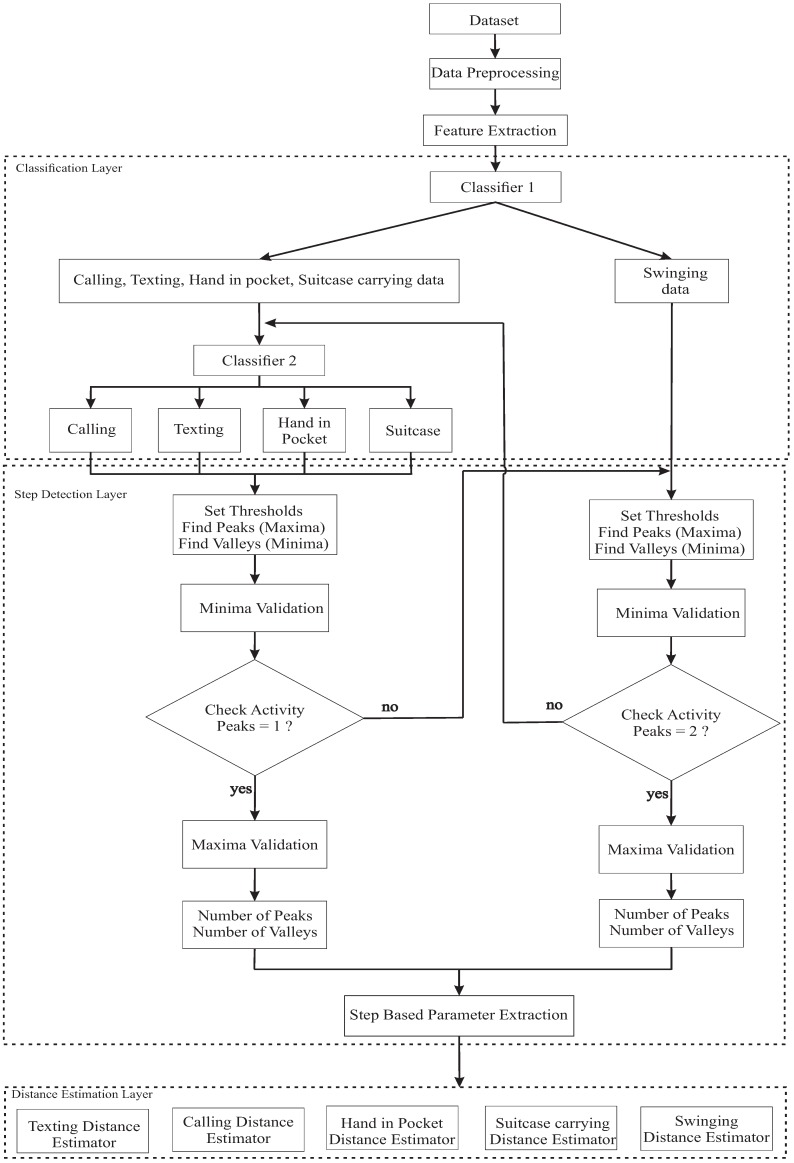
Proposed hierarchical framework of walking distance estimation.

The complete hierarchical framework of the walking distance estimation is illustrated in Figure 4. This framework is discussed in Section 2.2, Section 2.3, Section 2.4, Section 2.5 and Section 2.6, in order to describe the new walking distance estimation algorithm based on activity recognition using a smart band.

### 2.2. Data Collection and Pre-Processing

In this study, a smart band (Microsoft band 2) that integrates a tri-axis accelerometer (ST-Microelectronics, LSM6DS2, Scottsdale, AZ, USA) was used to collect tri-axis acceleration data. Ten healthy people participated in the experiments: six men (aged 24–27; height 170 ± 15.0 cm; weight 70.0 ± 5.0 kg) and four women (aged 24–25; height 168 ± 5.0 cm; weight 50.0 ± 2.0 kg). They were requested to wear the smart band and perform five wrist activities: texting, calling, hand in pocket, suitcase carrying and swinging during walking. Each person was required to repeat 20 m of walking at different speed levels 28 times for each wrist activity. The collected dataset contained 280 trials for each activity and 1400 trials in total (approximate 40 min of walking for each person) and was sampled at 62.5 Hz (maximum sampling frequency of the Microsoft band). In the preprocessing phase, we resampled the raw data at 50 Hz. For the frequency component of human body and the energy during perform movements below 15 Hz [16,17], we applied the collected tri-axis acceleration data a low-pass filter (10th-order Butterworth filter) with a cut-off frequency of 15 Hz.

### 2.3. Feature Extraction

The filtered signal does not characterize the activities. Therefore, we must extract the features from the data that characterize different activities. In this paper, 23 features were extracted from a sliding window of 100 samples data points with 50% overlap from the filtered data. This selection of window size was proven to be the successful solution for activity recognition in a previous work [18]. The following features, which have been shown to be effective in human activity recognition [18,19,20,21], are used in the paper:

- Average Energy (AE) [20,22,23]: The energy of each axis of the triaxial acceleration sensor is calculated by summing the squared discrete FFT component magnitudes of the signal in a sliding window. The AE in the paper is the average energy value calculated in three axes.
- Signal Magnitude Area (SMA) [20]
  (1) $$SMA=\frac{1}{n}\sum_{i=1}^{n}\left(\left|a_{i}^{x}\right|+\left|a_{i}^{y}\right|+\left|a_{i}^{z}\right|\right),$$
  where *n* is the size of a sliding window; $a^{x}$, $a^{y}$ and $a^{z}$ are sample point the acceleration data on three axes, *x*, *y* and *z*, of the triaxial sensor, respectively.
- Intensity of Movement (IM) [20,24]:
  (2) $$IM^{\left\{x,y,z\right\}}=\frac{1}{n}\sum_{i=1}^{n}\left|a_{n-i+1}^{\left\{x,y,z\right\}}-a_{n-i}^{\left\{x,y,z\right\}}\right|,$$
- Mean:
  (3) $$\mu^{\left\{x,y,z\right\}}=\frac{1}{n}\sum_{i=1}^{n}a_{i}^{\left\{x,y,z\right\}},$$
- Standard deviation [19,20,22]:
  (4) $$\sigma^{\left\{x,y,z\right\}}=\sqrt{\frac{1}{n}\sum_{i=1}^{n}\left(a_{i}^{\left\{x,y,z\right\}}-\mu^{\left\{x,y,z\right\}}\right)},$$
- Band power and peak power: the band power, which is defined as the power ratio in three frequency ranges (0–0.5 Hz, 0.5–1 Hz, 1–5 Hz), and the peak power, which is defined as the total power of the five dominant frequencies, are also effective features as demonstrated in [18]. The power in the band frequency from $f_{a}$ Hz to $f_{b}$ Hz is calculated by the following equation:
  (5) $$p=\frac{f_{b}-f_{a}}{2N}\left(S_{X}\left(f_{a}\right)+2\sum_{k=1}^{N}S_{X}\left(f_{k}\right)+S_{X}\left(f_{b}\right)\right),$$
  where $S_{X}\left(f\right)$ is the power spectral density of the Fourier transform of the acceleration signal; *N* is sampling frequency.

### 2.4. Activity Classification

In the activity classification task, two support vector machine (SVM) classifiers are used to classify five daily wrist activities during walking (Figure 4), since it is robust and highly accurate as demonstrated in other studies [18,25]. The first classifier is a binary SVM; it classifies two classes: swing activity and the other four activities. The second classifier is a multi-class SVM; it classifies four classes: texting, calling, hand in pocket and suitcase carrying. To select features for each classifier, we visualized the separation of wrist activities in the feature space. The corresponding feature of the classifiers are described in Table 1.

### 2.5. Step Event Detection

#### 2.5.1. Peak and Valley Detection

**Figure 5:**
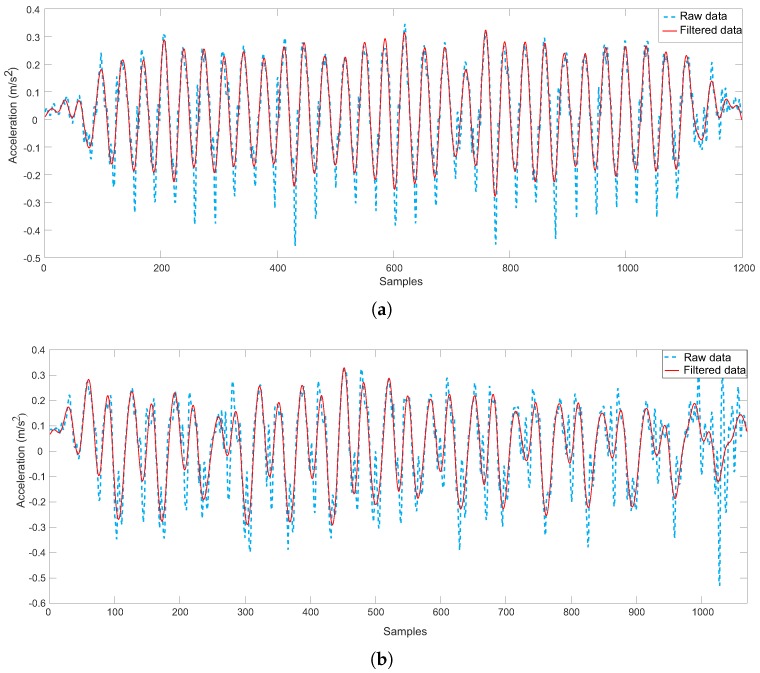
Raw and filtered acceleration data: (**a**) calling; (**b**) swinging.

After the hand motion mode is classified, the acceleration data are low-pass filtered again with a cut off frequency of 5 Hz to remove noise and avoid the failure in peak detection (Figure 5).

**Figure 6:**
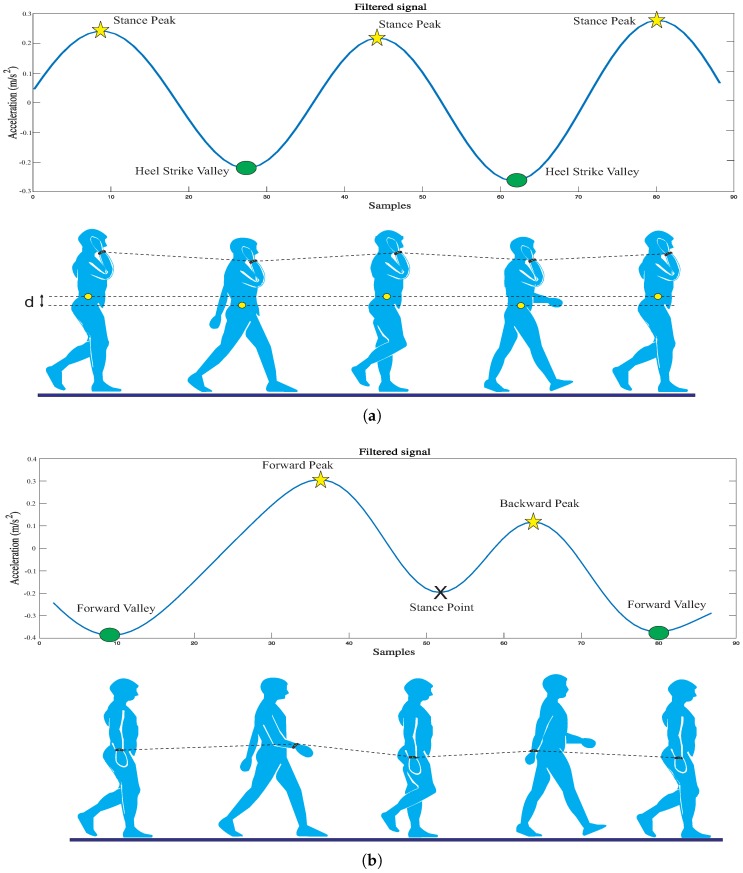
Relation between the vertical acceleration and the activities during walking: (**a**) calling; (**b**) swinging.

Park et al. [26] demonstrated that the arm and foot movements were synchronized during walking. Using this relationship, the step events are more easily detected by analyzing the collected acceleration data from the smart band. Figure 6 describes the insight into the wrist acceleration and arm movement. For the arm swinging, when the arm position is beyond or behind the user’s hip, the wrist’s acceleration value is maxima (peak); when the arm’s direction is perpendicular to the ground and tends to move forward, the wrist’s acceleration is minima (valley). The second case includes texting, calling, hand in pocket and suitcase carrying, whose common property is the center-of-mass movement. The acceleration in this case changes in a sinusoidal pattern because of the up and down motion of the user’s torso [9]. Therefore, the step detection problem can turn in the peaks and valleys detection of the acceleration signal of the wrist. The main difference between these two mentioned cases is the number of peaks between two valleys. In arm swinging, there are two peaks between two valleys, which corresponds to the number of steps. In other cases, there is only one peak between two valleys.

To detect the peaks and valleys of the wrist acceleration signal, we define the peak and valley thresholds. In addition, to minimize the probability of the miss-detection peaks and valleys, we initialize these values as follows:

(6) $$\mathrm{arm}\;\mathrm{swing}\;\mathrm{case}\;\left\{\begin{array}{c} th_{p}=0.5\times \max\left(a\right),\\ th_{v}=0.7\times \min\left(a\right), \end{array}\right.$$

(7) $$\mathrm{the}\;\mathrm{other}\;\mathrm{cases}\;\left\{\begin{array}{c} th_{p}=0.5\times \max\left(a\right),\\ th_{v}=0.5\times \min\left(a\right), \end{array}\right.$$

where $th_{p}$ and $th_{v}$ are the threshold values for the peak and valley detection, respectively; a is the vector of acceleration in a sample.

#### 2.5.2. Minimum Correction

The initialization of the thresholds is not the perfect value to detect the valleys (peaks) because some data do not clearly reflect the human action. Therefore, there are fewer detected valleys (peaks) than the actual valleys (peaks) or vice versa, which causes an incorrect detection of the actual steps. To resolve this issue, we define an abnormal interval $Ab_{in}$, which is the minimal distance between two valleys and calculated in Equation (9).

(8) $$\mu_{d}=\frac{1}{n_{v}-1}\times \sum_{i=1}^{n_{p}-1}(v_{i+1}-v_{i}),$$

(9) $$Ab_{in}=1.3\times \mu_{d}.$$

**Figure 7:**
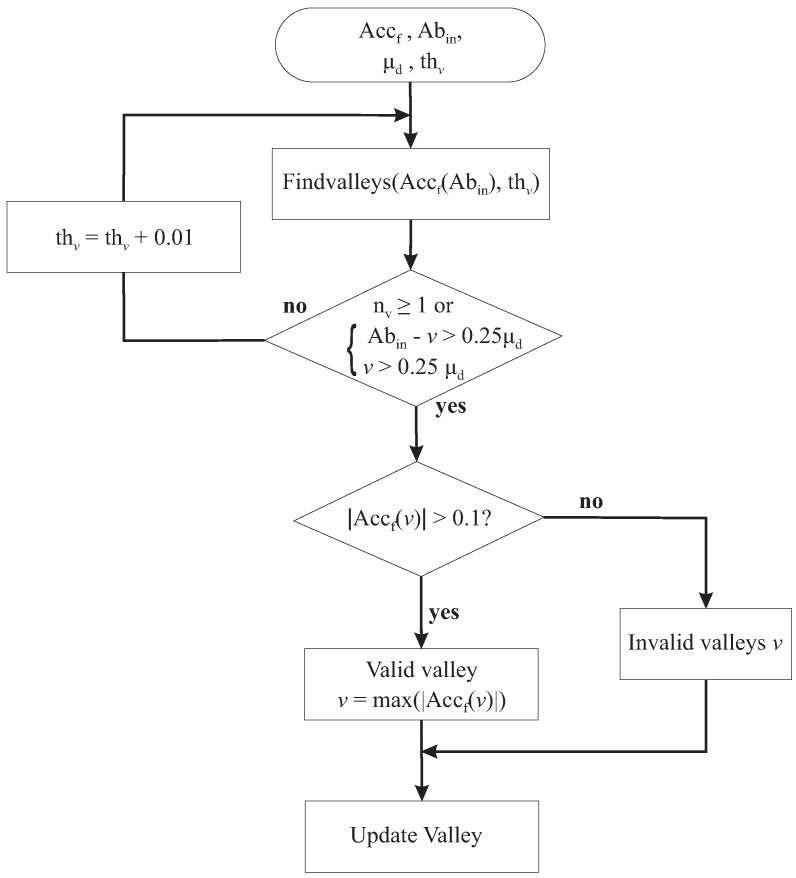
Minimum correction.

Any greater distance between two adjacent detected valleys than the abnormal interval is considered a missing valley in that interval. Those valleys will be detected again using our minimum correction algorithm with an adaptive threshold as illustrated in Figure 7. The notation in our algorithms is described in Table 2.

First, the Findvalleys function will take the abnormal interval (Equation (9)) and the threshold values for valleys (Equations (6) and (7)) as its input. The valley thresholds are increased in one of two scenarios: 1st—No valley is detected; 2nd—The distance between detected valleys to the first and to the end data point of abnormal interval smaller than a quarter of $\mu_{d}$. A valley will be considered invalid if the absolute value of the acceleration data at that valley is smaller than 0.1 m/s${}^{2}$ (considered a noise). If more than one valley is detected, the valley with the largest corresponding absolute acceleration is accepted.

#### 2.5.3. Missclassification Activity Feedback and Maximum Correction

Once all the valleys have been successfully detected, each interval between two adjacent valleys is considered a reference interval. As mentioned in Section 2.5.1, the number of peaks between two valleys is the utility information to contribute to the wrist’s activity classification. Therefore, we check the number of peaks in the reference interval. If the activities are classified as hand swing, but the number of reference intervals with two peaks exceeds 50% of the total reference intervals in one observation, the activities are considered as belonging to another wrist class activity. These activities will be fed back to the second classification layer as its input. Otherwise, if the activities are classified as belonging to group 2, but the number of reference intervals with one peak exceeds 50% of the total reference intervals of one observation, then it belongs to the hand-swinging class. These activities will be returned to the step detection algorithm of the hand-swinging case as its input.

**Figure 8:**
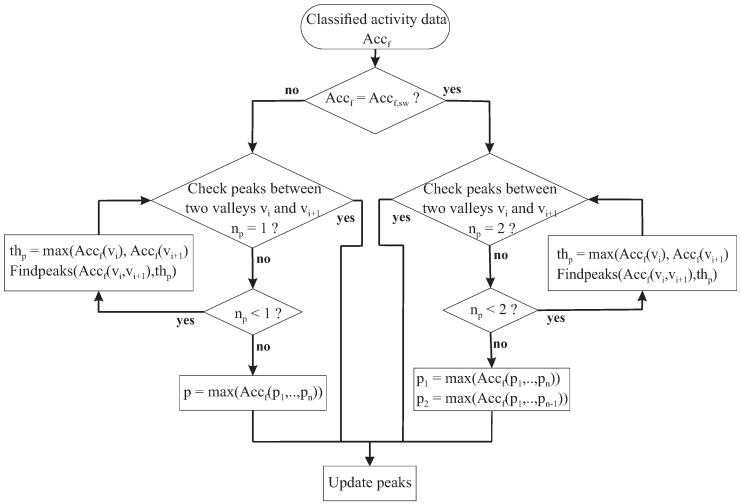
Maximum correction.

Capturing the step events or peak detection is the main factor to having a high accuracy of the distance estimation. Therefore, a maximum correction algorithm is necessary to correct the peaks that fail in the first detection, as described in Section 2.5.1. This algorithm is shown in Figure 8.

The key idea is to use all successful detected valleys and characteristics of the acceleration data for each type of activity during walking (analyzed in Section 2.5.1) to find the peaks that failed in the first detection times—the reference intervals in which incorrect peaks detection are taken into account. If there are two fewer peaks in the reference interval (in the swinging case, or one for the other cases), the Findpeaks function will detect the peaks one more times, where the threshold is the maximum acceleration data of the valleys in that interval. This threshold varies depending on the valleys of each reference interval to ensure that the peaks are successfully detected. In some cases, an irregular motion occurs during walking and causes redundant peaks to be detected. Then, two peaks (in the swinging case) or one peak (for the other case) with maximum accelerations are selected as the correct peaks.

### 2.6. Distance Estimation Method

For the walking distance estimation problem, a popular strategy is to sum up the length of all steps walked [13,27,28,29,30]. In the proposed method, we derive the equations of length steps based on the results of three previous studies [13,29,30]. These studies use a *K*-factor that is manually set according to the statistics of the volunteers. In the previous work [10], the *K*-factor was presented as a parametric model of polynomial regression. However, the parametric model is less robust and less flexibile than the non-parametric model [31]. Considering this issue, we propose the *K*-factor as a non-parametric regression model of the velocity features, which is called locally weighted polynomial regression [32,33]. Furthermore, we consider three step length equations of [13,29,30] to obtain the efficient distance estimators for each activity. The equations of the step length estimation are as follows:

- Weinberg method [13]:
  (10) $$L_{w}\approx \sqrt[{4}]{A_{\max}-A_{\min}}\times K,$$
  where $A_{\max}$ and $A_{\min}$ are the maximum and minimum accelerations in the vertical movement of the human body axis, and *K* is a constant unit for conversion (i.e., feet or meters traveled).
- Kim method [29]:
  (11) $$L_{k}=K\times \sqrt[{3}]{\frac{\sum_{i=1}^{N}\left|A_{i}\right|}{N}},$$
  where $A_{i}$ is the measured acceleration of sample *i*th in a single step; *N* is the number of samples covered in each step; and *K* is a constant unit for conversion.
- Tian method [30]:
  (12) $$L_{t}=K\times h\times \sqrt{f_{s}},$$
  where *h* is the height of the subject and $f_{s}$ is the step frequency, which is measured during the walking experiment; *K* is a constant unit for conversion.

Based on these methods, we derive the *K*-factor as a polynomial function of the step velocity:

(13) K=f(V,β)+e,i=1,2,...,n,

where

(14) $$V=\left[\begin{array}{cccc} 1 & \bar{v_{1}} & \cdots & {\bar{v_{1}}}^{p}\\ 1 & \bar{v_{2}} & \cdots & {\bar{v_{2}}}^{p}\\ \vdots & \vdots & \ddots & \vdots \\ 1 & \bar{v_{n}} & \cdots & {\bar{v_{n}}}^{p} \end{array}\right],$$

(15) $$K={\left(K_{1},K_{2},...,K_{n}\right)}^{T},$$

(16) $$\beta ={\left(\beta_{0},\beta_{1},...,\beta_{p}\right)}^{T},$$

(17) $${\begin{array}{c} e=(e_{1},e_{2},...,e_{n}), \end{array}}^{T}$$

where $\bar{v}$ is as the magnitude of the average velocities on three axes in each step; *e* and *n* are the noise and number of observations, respectively. We assume that *e* contains uncorrelated, mean zeros, and random variables [32]. Then, the problem is obtained by solving the weighted least-square problem:

(18) $$\min_{\beta }{\left(K-V\beta \right)}^{T}W\left(K-V\beta \right),$$

where *W* is a diagonal matrix with the Gaussian weight function, which can achieve a more accurate local approximation model and a smooth fit [34].

The solution for coefficient β is:

(19) $$\hat{\beta }={\left(V^{T}WV\right)}^{-1}\left(V^{T}WK\right).$$

The *K*-factor of the *i*th step is obtained as:

(20) $$K_{i}=\sum_{j=0}^{p}{\bar{v_{i}}}^{j}\beta_{j}.$$

The proposed adaptive step-length estimation equations are derived from three mentioned equations as follows:

Non-parametric Weinberg method:

(21) $$L_{step_{i}}=\sum_{j=0}^{p}{\bar{v_{i}}}^{j}\beta_{j}\times \sqrt[{4}]{A_{max}-A_{min}},$$

Non-parametric Kim method:

(22) $$L_{step_{i}}=\sum_{j=0}^{p}{\bar{v_{i}}}^{j}\beta_{j}\times \sqrt[{3}]{\frac{\sum_{k=1}^{N}\left|A_{k}\right|}{N}},$$

Non-parametric Tian method:

(23) $$L_{step_{i}}=\sum_{j=0}^{p}{\bar{v_{i}}}^{j}\beta_{j}\times h\times \sqrt{f_{s}}.$$

The walking distance is calculated by summing all steps for each experiment:

(24) $$D=\sum_{i=1}^{N}L_{step_{i}},$$

where *N* is the number of walked steps in each experimental sample.

## 3. Experimental Results

### 3.1. Activity Classification

As mentioned, to collect sufficient data to assess the performance of our proposed method, ten participants were requested to perform five daily wrist activities in 20 m of walking at different levels of speed. We used a confusion matrix to estimate the performance of the classifiers in Table 3 (classifier 1) and Table 4 (classifier 2). As described in these tables, the first column lists the performed activities by the participants, and the first row lists the predicted activities by the classifiers.

In Table 3, the swing activity is 100% correctly predicted. As mentioned, the swing acceleration data is significantly different from other cases. In addition to the up and down actions of the hip, the forward and backward actions of the arms also affect the acceleration data. This characteristic makes the swinging activity different from the other activities. The accuracy of predicting texting/calling/hand in pocket/suitcase carrying is 99%. The first classifier incorrectly predicted 1% of them as swinging. Texting, calling, hand in pocket, and suitcase carrying are center-of-mass motions, and the acceleration is generated by the up and down actions of the hip, but, in some cases, the arm slightly moves because of the inertia of fast walking. In this situation, texting/calling/hand in pocket/suitcase carrying is identical to swinging at a slow speed, so the classifier failed to classify these activities. All activities that are predicted as swinging are the input of the swinging step detector. The 1% incorrectly predicted activity is rechecked in the step detector and returned to the second classifier.

The classes (texting, calling, hand in pocket and suitcase carrying) from the first classifier are the input of the second classifier. The confusion matrix is provided in Table 4. The hand in pocket is perfectly predicted. The calling and suitcase carrying are 2% incorrectly predicted as texting and calling, respectively. The texting is 1% incorrectly classified as hand in pocket. Those errors affect the performance of the step detector but in acceptable amounts because all activities are one peak between two valleys.

### 3.2. Step Detection

**Figure 9:**
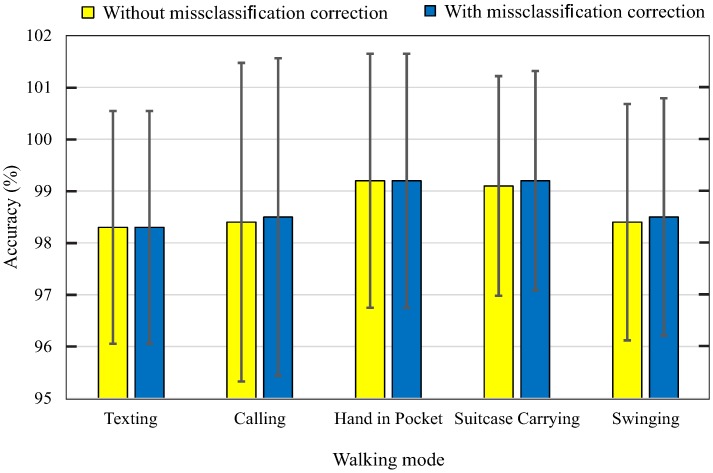
Step detection performances.

Classified data are fed to the step detector, which has five different reference and adaptive thresholds for five walking activities. The misclassifications of the first classifier are returned and corrected. The step detection algorithm is affected by the wrong classification of the first and second classifier. Figure 9 illustrates the accuracy and standard variance of the step detection between with and without misclassification correction for each walking activity. As shown in the figure, the accuracy of the step detection algorithm with misclassification correction is higher than without misclassification correction in the calling, suitcase carrying and swinging. This is because the 1% error of predicted swinging activity in the first classifier is returned to the second classifier. For step detection with misclassification correction, the accuracy of each walking activity is higher than 98% and the highest standard deviation is 3%.

We must emphasize that, for each walking activity, the vertical acceleration data change among the *x*, *y* and *z*-axes. It is difficult for the step detection and distance estimation algorithm when we use the vertical acceleration data as a fixed axis. The classification data solve these problems by classifying the activity and using corresponding vertical data of that activity. In addition, the adaptive threshold also renders the step detection performance.

### 3.3. Walking Distance Estimation

To evaluate the performance of the proposed method, the Leave-One-Sample-Out technique, which makes one trial a test set and the remaining trials the training set in each epoch, was applied to the classified activity data. This technique is commonly used for small datasets [35]. We derived the *K*-factor as a *p*-degree polynomial function of the velocity feature. In the experiment, we examined various values of *p* to minimize the estimation error. The polynomial degree *p* of the *K*-factor, which was implemented for three methods [13,29,30], was four.

The walking speeds are: low speed ($v\leq \bar{v}-\sigma$), normal speed ($\bar{v}-\sigma <v<\bar{v}+\sigma$) and high speed ($\bar{v}+\sigma <v$). Here, $\bar{v}$ is the average speeds and σ is the deviation of human walking speed [10]. The performance (accuracy, standard deviation (Std) and normalized mean square error (NMSE)) of each traveling distance estimator considering the activities and walking speed is presented in Table 5.

All three proposed methods estimate the walking distance in the texting activity efficiently, and the performance is best when the person walks at high speed (the accuracy is more than 97.91%). The average distance accuracy is higher than 97% for low, normal and high speeds, the normalized mean square error is 1.19, and the standard variance is acceptable (below 0.92 m). Otherwise, with the calling case, the estimated distance at high walking speed is lower than that at normal and low walking speeds. For the other case, the accuracy does not depend on the walking speed but on the deployed method. For example, in the hand-in-pocket case, the highest accuracy is 97.89% using the non-parametric Kim method at high walking speed, and the worst accuracy is 93.33% using the non-parametric Tian method at low speed. The swinging case has a larger standard variance than the other activities due to the change in vertical acceleration as a result of both arm swinging and hip moving during walking.

One of our objectives is to select the best distance estimators that can be stable and achieve high accuracy for each hand daily activity using a smart band. The non-parametric Tian estimator was implemented to estimate the walking distance of texting, calling, and suitcase-carrying activities. In addition, the non-parametric Kim estimator was used for the hand-in-pocket and swinging activities.

According to Table 6, the proposed method achieved an average accuracy of 96.9%, whereas that of the reference method is 95.1%. The calling-during-walking experiment is the most unstable because of different arm gestures of phone call and arm fatigue during the experiments. The smallest and largest gaps of accuracy between the proposed method and reference method were found for the texting and hand in pocket activities, respectively.

**Figure 10:**
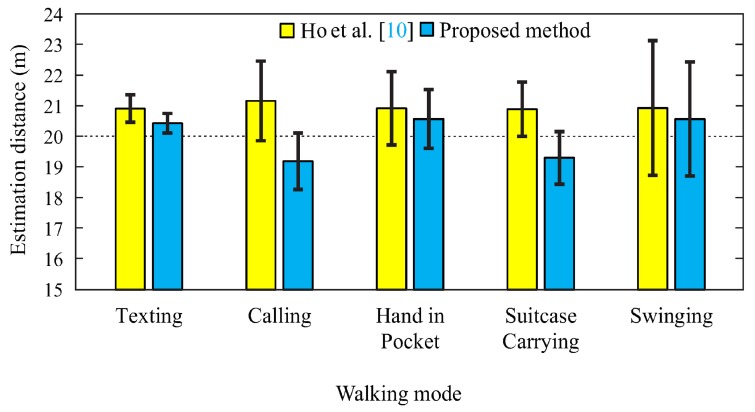
Performance comparison of the proposed method and the reference method.

According to Figure 10, the proposed and reference methods suffer low accuracy and high standard deviation for the suitcase-carrying and calling activities, respectively. Overall, an aspect of the proposed method that used walking distance estimators with each activity can surpass the reference method in terms of accuracy and standard deviation.

## 4. Conclusions

In this paper, a step detection algorithm and a walking distance estimation based on daily hand activity recognition using the smart band have been presented and experimentally evaluated. Five daily hand activities during walking were considered: phone calling, phone texting, hand in pocket, suitcase carrying and swinging. Each hand activity has different vertical acceleration data, and changing the vertical acceleration data of the smart band is the main challenge of the distance estimation. Therefore, two SVM classifiers are used to classify and let the step detector and distance estimator know the activity that is processed. In addition, the classification is processed in two steps to improve the robustness of the step detection and walking distance estimation by feedback data of the wrong candidates. The new step detection and distance estimation algorithm using the smart band have been presented. To evaluate the performance of the proposed method, experiments of 20-m walking while performing daily hand activities using the Microsoft smart band were conducted with ten participants. The accuracy of this classification was above 99% for all activities of both classifiers. With prior knowledge about the data being processed, the adaptive threshold strategy of the step detection algorithm is effectively performed. The error of misstep detection is approximately 2%. The experiment results also show the performance of three non-parametric methods, and we compared the performance of the walking distance estimation algorithm with the reference method. The result shows that the proposed method has outstanding accuracy and robustness.

In the proposed method, a post-hoc analysis has been applied. For real applications, real-time processing algorithms will be required. Also, to enhance the performance of estimation, we should consider other daily living activities. These remain future work.

## Figures and Tables

**Table 1:** Corresponding features of the classifiers.

Classifier	Feature
SVM 1	SMA, IM, AE, Band power, Peak power
SVM 2	μ, σ, AE, Band power, Peak power

**Table 2:** Notation of the variables used in the algorithms.

Notation	Description
$Acc_{f}$	Filtered and classified acceleration signal
$Acc_{f,sw}$	Filtered and Classified acceleration signal of swinging
$Ab_{in}$	Abnormal interval of one observation
$th_{p}$	Adaptive peak threshold
$th_{v}$	Adaptive valley threshold
$n_{p}$	Number of detected peak
$n_{v}$	Number of detected valley
*p*	Detected peak position in a sample data
*v*	Detected valley position in a sample data
*d*	Distance between two detected valleys
$\mu_{d}$	Mean of distances between two detected valleys

**Table 3:** Classification results: swinging versus texting, calling, hand in pocket and suitcase carrying.

Activity	Predicted Class	
	Swinging	Texting/Calling/Hand in Pocket/Suitcase Carrying
Swinging	**100%**	0%
Texting/Calling/Hand in Pocket/Suitcase Carrying	1%	99%

**Table 4:** Classification results: texting, calling, hand in pocket, suitcase carrying.

Activity	Predicted Class			
	Texting	Calling	Hand in Pocket	Suitcase Carrying
Texting	**99%**	0%	1%	0%
Calling	2%	**98%**	0%	0%
Hand in Pocket	0%	0%	**100%**	0%
Suitcase Carrying	0%	2%	0%	**98%**

**Table 5:** Distance estimation accuracy of the proposed method.

Activity	Walking Speed	Proposed Distance Estimation Method					
		Non-Parametric Weinberg Method		Non-Parametric Kim Method		Non-Parametric Tian Method	
		Accuracy(%)	Std(m)	Accuracy(%)	Std(m)	Accuracy(%)	Std(m)
**Texting**	Low	97.25	0.45	96.84	0.71	97.65	0.32
	Normal	95.80	0.92	96.44	0.46	97.82	0.43
	High	98.74	0.40	98.35	0.45	97.91	0.21
	NMSE	1.27		1.31		1.19	
	**Average accuracy**	97.26		97.21		**97.79**	
**Calling**	Low	94.81	0.84	95.25	0.85	96.79	0.79
	Normal	95.37	1.06	95.42	1.06	95.47	1.04
	High	94.72	1.50	94.45	0.93	95.12	0.93
	NMSE	1.02		1.10		1.01	
	**Average accuracy**	94.96		95.04		**95.79**	
**Hand in Pocket**	Low	93.36	0.86	96.34	0.83	93.33	1.80
	Normal	96.96	0.67	97.12	0.67	96.98	0.67
	High	96.09	0.72	97.89	1.38	96.15	1.23
	NMSE	2.24		1.96		1.96	
	**Average accuracy**	95.47		**97.11**		95.49	
**Suitcase Carrying**	Low	95.71	1.29	96.46	1.12	97.73	1.27
	Normal	96.54	0.89	96.52	0.87	96.53	0.82
	High	96.34	0.41	94.67	0.42	95.38	0.46
	NMSE	2.64		2.60		2.10	
	**Average accuracy**	96.20		95.89		**96.54**	
**Swinging**	Low	94.21	2.84	96.47	2.67	95.10	0.70
	Normal	96.59	1.75	97.26	1.68	97.22	1.68
	High	94.87	1.26	97.56	1.23	94.13	1.23
	NMSE	1.80		2.44		1.20	
	**Average accuracy**	95.23		**97.09**		95.45	

**Table 6:** Accuracy of the distance estimation of the proposed method and the reference method.

Activity	Ho et al. [10]	Proposed Method
	Accuracy(%)	Accuracy(%)
Texting	95.44	97.79
Calling	94.16	95.79
Hand in Pocket	95.35	97.11
Suitcase Carrying	95.02	96.54
Swinging	95.38	97.09
